# Crafting Careers: Unraveling the Impact of Career Crafting on Career Outcomes and the Moderating Role of Supervisor Career Support Mentoring

**DOI:** 10.3390/bs15060740

**Published:** 2025-05-27

**Authors:** Anguo Fu, Shuaihua Wang, Xinyao Gan, Shenyang Hai

**Affiliations:** International Business School, Hainan University, Haikou 570228, China; fuanguo@hainanu.edu.cn (A.F.); 22211202020003@hainanu.edu.cn (S.W.); 24211202020001@hainanu.edu.cn (X.G.)

**Keywords:** career crafting, sense of control, career engagement, career turnover intentions, supervisor career support mentoring

## Abstract

With rapidly advancing artificial intelligence and digital transformation, career development is becoming increasingly uncertain and complex. A key strategy for individuals to adapt to dynamic environments is career crafting; however, research on how employees use it to enhance their career outcomes remains limited. Based on the conservation of resources theory and career construction theory, this study posits that career crafting enhances employees’ sense of control by helping them cope with career and environmental changes. This increased sense of control fosters greater career engagement and reduces career turnover intentions. Furthermore, we examine supervisor career support mentoring as a key contextual element shaping career crafting effectiveness. We collected three-wave data at one-week intervals from 232 hospitality frontline employees. Employees reported their career crafting, supervisor career support mentoring, and demographic information at Time 1; sense of control at Time 2; and career engagement and career turnover intentions at Time 3. The results indicate that career crafting improves employees’ sense of control, which enhances career engagement and reduces career turnover intentions. Additionally, supervisor career support mentoring strengthens the indirect effect of career crafting on career engagement and career turnover intentions through its impact on the sense of control. This study enriches our understanding of career crafting and of effectively leveraging its positive effects in organizational management.

## 1. Introduction

Artificial intelligence (AI) technology accelerates innovation and deepens digital transformation, resulting in career development becoming increasingly complex and uncertain (Bankins et al., 2024). In the hospitality industry, frontline employees face various challenges, including rigid career paths and limited promotion opportunities, with their career growth often constrained by industry-specific characteristics (e.g., rapid skill iteration and high turnover) (Leong et al., 2024). Introducing AI-driven automation in service models, such as intelligent customer service systems and robot receptionists, further intensifies career insecurity among frontline employees by threatening traditional career trajectories (Cai et al., 2024; Steindórsdóttir et al., 2024). This technological shift has made it essential for employees to move beyond passive career pathways and proactively shape their careers. In this context, “career crafting” has emerged as a bottom-up career design approach that serves as a key strategy for individuals to navigate dynamic environments (Alarifi et al., 2024; Diaa et al., 2024; Ge et al., 2023; Tims & Akkermans, 2020). Rather than passively adhering to a fixed career path, career crafting enables employees to actively pursue their desired career trajectories (Leong et al., 2024; Ramaprasad et al., 2022). Career crafting encompasses proactive behaviors by which individuals self-manage their careers, with the goal of achieving the optimal person–career fit (Tims & Akkermans, 2020, p. 176). By adjusting career goals, skill development, and networking, career crafting not only plays a crucial role in strengthening employees’ job engagement, job performance, and employability but also reduces turnover intentions, improving both individual and organizational sustainability (Diaa et al., 2024; Kilic & Kitapci, 2024; J. Y. Lee et al., 2021; Leong et al., 2024; Tims & Akkermans, 2020). Given the increasing complexity of career development in the face of digital transformation and AI-driven changes, understanding how and when career crafting influences employees’ career outcomes has become a critical issue in both academic and practical contexts.

While previous research made some progress, our understanding of career crafting remains limited. First, the existing research has concentrated primarily on examining the effects of job crafting on both employees and organizations, neglecting the significance of career crafting in shaping employee behavior and organizational development. While both are self-initiated behaviors, job crafting involves employees making modifications to better align job demands and available resources with their individual abilities and personal requirements, thus enhancing the person–job fit (W. Li et al., 2024a; Tims et al., 2012). By comparison, career crafting emphasizes the congruence between individuals and their overall career trajectories, encompassing a broader range of career-related behaviors (Leong et al., 2024; Tims & Akkermans, 2020). As contemporary careers become increasingly dynamic and flexible, individuals must actively shape and strategically design their career trajectories to create a sustainable and satisfying career journey rather than follow a fixed, stable path within one organization (Akkermans & Tims, 2017; De Vos et al., 2019; Janssen et al., 2021). Consequently, elucidating the consequences of career crafting for employees is essential. Second, while prior studies have investigated the impact of career crafting across various career outcomes (J. Y. Lee et al., 2021; Leong et al., 2024; Tims & Akkermans, 2020), the mechanisms through which career crafting influences these outcomes and the factors that amplify or mitigate its effects remain underexplored. This theoretical gap limits our ability to develop effective interventions in practice, particularly in high-turnover industries such as hospitality, where a systematic framework is urgently needed to guide employees’ career development.

To bridge this theoretical gap and address practical challenges, this study draws on conservation of resources (COR) theory and career construction theory (CCT) to examine how and under what conditions career crafting influences employees’ career engagement and career turnover intentions. Career engagement refers to the process through which individuals acquire and invest resources throughout their careers; it plays a critical role in enhancing long-term career development and job satisfaction (Upadyaya & Salmela-Aro, 2015). Career turnover intentions are a defensive response to resource depletion, in which individuals assess potential resource losses amid career uncertainty (Barthauer et al., 2020). The roles of career engagement and career turnover intentions are vital in dynamic career environments, especially in high-turnover sectors such as hospitality, where engagement serves as a key indicator of service effectiveness, and turnover intentions are major predictors of organizational sustainability (Bufquin et al., 2021; Hirschi et al., 2014; I. J. Park et al., 2020). This study, therefore, examined the influence of career crafting on career engagement and turnover intentions among frontline employees in the hospitality industry.

According to COR theory, individuals strive to acquire, maintain, and protect valuable resources (Hobfoll, 1989, 2001). Career crafting represents a proactive resource management behavior through which employees acquire, sustain, and leverage career-related resources to pursue their professional aspirations and enhance their well-being (Janssen et al., 2021; Leong et al., 2024). In the face of increasing uncertainty and complexity in career development, employees experience heightened career insecurity (Bankins et al., 2024; Cai et al., 2024), which can lead to significant resource depletion (Shi et al., 2022). COR theory’s resource gain paradox posits that resource acquisition becomes especially critical when individuals are confronted with resource loss (Breevaart & Tims, 2019; Hobfoll et al., 2018). To preserve their well-being and mitigate potential losses, employees may engage in resource-protective behaviors aimed at replenishing their resources (Harju et al., 2021; Hobfoll, 1989; Lu et al., 2022). Specifically, when faced with career insecurity and instability, individuals may turn to career crafting as a means to manage and replenish their resource pool. Simultaneously, they are motivated to reinvest their existing resources to safeguard what they already possess (Harju et al., 2016; Hobfoll et al., 2018; Meijerink et al., 2020). Through career crafting, such as proactive career reflection and proactive career construction, employees strategically manage and utilize their resources to develop additional ones, thereby expanding their resource reservoir and supporting career goal attainment (Leong et al., 2024; Lu et al., 2022). This process enhances their sense of control, a critical cognitive resource that not only facilitates further resource investment but also initiates a gain spiral, thereby promoting increasingly favorable career outcomes (Hobfoll et al., 2018). These outcomes include greater career engagement and reduced career turnover intentions.

In addition, drawing on CCT (Savickas, 2005), we conceptualize career crafting as a form of individual adaptive readiness. CCT explains how individuals actively shape their career development through meaningful vocational behaviors and work experiences within a dynamic and evolving context (Leong et al., 2024; Savickas, 2013; Wehrle et al., 2019). According to the theory, career construction follows a logical sequence from adaptive readiness, through adaptive resources, to career adaptability outcomes (Seibert et al., 2021). We argue that career crafting, as a reflection of employees’ adaptive readiness, enables individuals to enhance their adaptive resources, particularly their sense of control. Within this framework, increased career engagement constitutes a key adaptability outcome, while reduced career turnover intentions serve as another. Despite its theoretical relevance, limited research has explored career crafting as an intentional strategy for responding to career-related threats, maintaining or gaining resources, or as a precursor to adaptability outcomes. Therefore, integrating COR theory with career construction theory, we propose that a sense of control functions as a central mediating mechanism linking career crafting to both career engagement and career turnover intentions.

Furthermore, according to COR theory, contextual factors play a crucial role in influencing the acquisition and preservation of resources (Hobfoll et al., 2018). CCT also underscores that individual career development is shaped by the dynamic interplay between a person and their environment (Savickas, 2013). Responding to a research call (Tims & Akkermans, 2020), this study examined the extent to which significant others at work—particularly supervisors—influence employees’ career-crafting behaviors. In the workplace, supervisors frequently interact with employees and exert substantial control over the allocation of organizational resources (He et al., 2024; L. Wang et al., 2018), offering employees access to opportunities and resources for career development (Deng et al., 2023; Erdogan et al., 2025). Therefore, supervisor career support mentoring serves as a key contextual element that influences the effectiveness of employees’ career crafting. Specifically, when frontline employees perceive higher levels of supervisor career support mentoring, they typically have more career-related resources and autonomy to engage in career crafting, which strengthens its positive effects on their sense of control and subsequent career outcomes. Conversely, when employees perceive lower levels of supervisor career support mentoring, they may lack the necessary support, which limits their autonomy and opportunities for career crafting. This constraint may weaken the relationship between career crafting and career outcomes.

**Figure 1:**
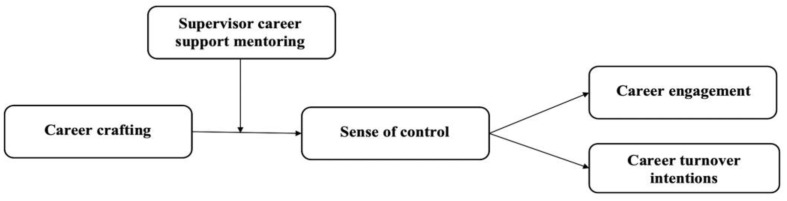
Hypothesized research model.

To test our research model (Figure 1), we employed a time-lagged design to collect data from frontline employees in the hospitality industry. This research design makes it possible to precisely capture the temporal sequence of variables and helps mitigate the effects of common method variance (CMV) (Podsakoff et al., 2024). This study offers two key theoretical contributions. First, it introduces a novel theoretical framework that explains how career crafting influences employees’ career outcomes, specifically career engagement and career turnover intentions. While previous studies have elucidated the consequences of career crafting (J. Y. Lee et al., 2021; Leong et al., 2024), few have examined the mechanisms that link career crafting to career engagement and career turnover intentions. By addressing this gap, this study expands the literature on career crafting. Second, it identifies supervisor career support mentoring as a critical contextual factor that enhances the effectiveness of career crafting. This insight clarifies the contextual factors that help career crafting produce desirable organizational outcomes, offering a more comprehensive understanding of how and when career crafting enhances employees’ career outcomes.

In practice, this research has substantial implications for high-turnover industries such as hospitality, where employee retention and engagement are persistent challenges (I. J. Park et al., 2020). Our findings suggest that career crafting can serve as an effective strategy for reducing career turnover intentions and improving career engagement among frontline employees. Moreover, supervisor career support mentoring plays a crucial role in enhancing the positive effects of career crafting, offering organizations a practical tool to support their employees’ career development. This study offers valuable insights for managers, helping them reduce employee turnover, improve service quality, and achieve long-term sustainable development for their organizations.

## 2. Literature Review and Hypothesis Development

### 2.1. Career Crafting, Conservation of Resources Theory, and Career Construction Theory

Career crafting encompasses proactive behaviors that help individuals self-manage their careers to achieve the optimal person–career fit (Tims & Akkermans, 2020). This construct operationalizes two core dimensions: proactive career reflection and proactive career construction. Proactive career reflection focuses on exploring and evaluating career-related motivations, values, and aspirations, while proactive career construction involves proactive behaviors related to interpersonal networks, self-analysis, and goal pursuit within career contexts (Janssen et al., 2021; Tims & Akkermans, 2020). Individuals who actively reflect on their career motivations and abilities while taking proactive steps to advance their career development through networking are more likely to achieve their career objectives (J. Y. Lee et al., 2021; Leong et al., 2024; Tims & Akkermans, 2020). Guided by COR theory, this study conceptualizes career crafting as a proactive resource management strategy through which individuals utilize, sustain, and acquire career-related resources to support their professional development (De Vos et al., 2020; Hirschi & Koen, 2021; Janssen et al., 2021). Prior research (e.g., Doden et al., 2024; Halbesleben et al., 2014; Meijerink et al., 2020; Yang et al., 2022) supports this perspective, showing that engaging in crafting behaviors is an effective way for individuals to access and preserve valuable resources. Specifically, employees engage in career crafting by exploring available career resources through proactive career reflection, which cultivates insight into their career trajectories (Janssen et al., 2021). In addition, they practice proactive career construction to articulate their strengths and professional goals, thereby leveraging and maintaining their professional networks—resources that, in turn, generate new career opportunities (Hirschi et al., 2018). Through career crafting, employees can strategically manage their resources, concentrate on their strengths, and build career-related competencies, ultimately improving career performance (Cheng & Yi, 2018) and reinforcing their sense of control.

In COR theory, a sense of control is considered a key resource (Hobfoll, 1989). Individuals with abundant resources gain new resources through investment, which fosters further resource growth, creating a gain spiral (Hobfoll et al., 2018). Moreover, employees with greater resources are more likely to invest those resources in work-related tasks, thereby building additional resources more effectively. Research shows that employees with a higher sense of control demonstrate better work and career performance (A. Li et al., 2021). Moreover, CCT posits that individuals can actively manage their career success and take personal responsibility for their vocational development (Savickas, 2005). Career crafting can be conceptualized as a form of adaptive readiness aimed at enhancing the person–career fit by strengthening adaptive resources and ultimately improving career adaptability outcomes. Consequently, those who strengthen their sense of control through career crafting are more likely to achieve better career outcomes. This research examined two key career outcomes—career engagement and career turnover intentions—of frontline employees in the hospitality sector, which is a high-turnover industry (Bufquin et al., 2021; Hirschi et al., 2014; Nilforooshan & Salimi, 2016). Specifically, career engagement reflects the degree to which an individual actively shapes their career through various behaviors (Hirschi et al., 2014). In contrast, career turnover intentions indicate the extent to which an individual considers exiting their profession or chosen career path (Barthauer et al., 2020). Therefore, this study posited that career crafting enhances employees’ sense of control, which, in turn, increases their career engagement and reduces career turnover intentions. In addition, a supportive social environment—such as supervisor career support mentoring—offers employees opportunities and space to acquire and expand resources, thereby providing fertile ground for the creation, maintenance, and management of resources that facilitate desirable career adaptability outcomes (Deng et al., 2023; Erdogan et al., 2025; Hobfoll et al., 2018; Leong et al., 2024; Savickas, 2013; Wehrle et al., 2019). Integrating COR theory and career construction theory (Hobfoll et al., 2018; Savickas, 2013), we further posited that the positive impact of career crafting on a sense of control and its subsequent effects may be strengthened by supervisor career support mentoring.

### 2.2. Career Crafting and Career Outcomes

The COR theory posits that individuals can accumulate resources through proactive behaviors, which subsequently enhances their ability to cope with environmental stressors (Hobfoll et al., 2018). This study proposes that career crafting, as a proactive resource management behavior, may facilitate resource generation, strengthening employees’ sense of control. A sense of control is defined as an individual’s general perception of their ability to shape their environment or be influenced by it (Sirola, 2024); it is considered an individual resource used to respond to environmental stress (Vander Elst et al., 2014). It reflects an individual’s capacity to efficiently interact with their surroundings, exhibit competence, and achieve desired outcomes (A. Li et al., 2021). Individuals with a heightened sense of control generally believe that they can influence their environment (A. Li et al., 2021; Sirola, 2024). Career crafting helps employees effectively cope with work and career changes, enhance their employability and career sustainability, and strengthen their sense of control over work situations. According to CCT (Savickas, 2005), individuals gain psychosocial resources and develop greater competence through proactive career behaviors, which enhance their ability to cope with changes and challenges in their work and careers (Zacher, 2015). Through career crafting, employees can benefit from accessing career-related information and resources, which, in turn, improves their capacity to solve problems and accomplish tasks (Leong et al., 2024). This process enhances their sense of control and contributes to favorable career outcomes.

Career crafters continuously plan their careers to align with their interests, needs, values, and strengths, actively taking control of their career direction rather than passively accepting external circumstances (J. Y. Lee et al., 2021; Tims & Akkermans, 2020). Through such proactive career behaviors, employees gain additional psychological and social resources, which enable them to successfully handle changes within their work and careers, such as involuntary and unpredictable career shocks (Akkermans & Hirschi, 2023; Janssen et al., 2021; Leong et al., 2024). This enhances their sense of control in the work environment. Second, through proactive career reflection and construction, employees enhance their employability and career sustainability (Akkermans & Hirschi, 2023). For example, employees reflect on their weaknesses and improve their professional skills through training and education. This helps them achieve more favorable career outcomes and better align their individual capabilities with their career (Ni et al., 2024; Tims & Akkermans, 2020), which fosters a stronger sense of control. Additionally, individuals engaged in career crafting actively expand their career-related resources and proactively seek career opportunities. This enhances their ability to attain expected professional and career achievements (Ge et al., 2023; J. Y. Lee et al., 2021), further contributing to their sense of control.

**Hypothesis 1.**  *Career crafting is positively related to a sense of control.*

According to COR theory, employees’ participation in career crafting increases their sense of control, which, in turn, motivates them to invest more resources into their careers in anticipation of further resource gains (Hobfoll et al., 2018). Thus, we posit that career crafting enhances employees’ sense of control, leading to higher career engagement. First, the sense of control derived from career crafting may strengthen employees’ belief that their actions will lead to positive career outcomes and improve their career prospects (Sirola, 2024; Tims & Akkermans, 2020). This belief not only enhances their motivation to develop human capital, making them more inclined to dedicate time, effort, and resources to their careers (Gabriel et al., 2020; A. Li et al., 2021) but also fosters positive self-perceptions and self-efficacy. Increased self-efficacy further encourages employees to exert effort at work to acquire additional resources (Hirschi & Freund, 2014). Second, as a key resource, a stronger sense of control can elevate employees’ overall resource levels. Those with greater control over their careers are more likely to receive both instrumental and emotional support from others (Infurna et al., 2016). Therefore, a sense of control, along with the resources it generates, enhances employees’ vitality and energy while strengthening their belief in their ability to influence their environment (Sirola, 2024). As ample resources, sustained energy, and a positive perception of the environment are key prerequisites for career engagement (Hirschi et al., 2014; Hirschi & Freund, 2014), we propose that a sense of control fostered by career crafting further promotes employees’ career engagement.

**Hypothesis 2a.**  *Career crafting indirectly affects career engagement through a sense of control.*

According to COR theory, acquiring a sense of control as a resource helps employees cope with career-related stressors, mitigates their negative effects, and enhances well-being (Hobfoll, 2001). First, a higher sense of control gained through career crafting improves employees’ emotional regulation, making them more inclined to use problem-focused coping strategies (Infurna et al., 2016). When faced with career challenges, it helps employees better manage their negative emotions and maintain optimism (A. Li et al., 2021). Conversely, when employees experience resource depletion and perceive a lack of control over stressful situations, they tend to rely on emotion-based coping strategies, such as avoidance and withdrawal (Lazarus & Folkman, 1984; Vander Elst et al., 2014). This can heighten their career turnover intentions (Barthauer et al., 2020). For instance, even when individuals recognize barriers to career advancement and limitations to career growth, career crafting allows them to seek coping strategies that enhance their sense of control over their career development, which reduces career turnover intentions. Second, a sense of control helps employees release emotional energy, preventing persistent emotional rumination (Gabriel et al., 2020; Lazarus & Folkman, 1984), which reduces the negative impact of adverse career events. Furthermore, a sense of control strengthens employees’ organizational commitment while reducing psychological withdrawal (Elst et al., 2011; Vander Elst et al., 2014), enhancing their connection to the organization and lowering their career turnover intentions. Employees with a strong sense of control exhibit weaker tendencies toward leaving their organization (X. Li et al., 2020). Consequently, we propose that the sense of control derived from career crafting further alleviates employees’ career turnover intentions.

**Hypothesis 2b.**  *Career crafting indirectly affects career turnover intentions through a sense of control.*

### 2.3. The Moderating Role of Supervisor Career Support Mentoring

According to COR theory, a supportive social environment plays a critical role in helping employees acquire, protect, and sustain resources (Hobfoll et al., 2018). CCT similarly emphasizes that individuals’ career development is shaped by the dynamic interaction between personal agency and contextual factors (Savickas, 2013). As direct managers, supervisors have frequent opportunities to interact with employees and exert significant influence over the distribution of organizational resources (L. Wang et al., 2018). Therefore, integrating COR theory and CCT, supervisor career support mentoring is regarded as a key contextual factor in shaping the outcomes of employees’ career-crafting behaviors (Erdogan et al., 2025). This mentoring involves offering employees opportunities for skill development and networking, as well as access to challenging tasks (Deng et al., 2023; Y. Wang et al., 2024). It plays a crucial role in ensuring the effectiveness of career crafting, as it provides the necessary resources needed for career development (Deng et al., 2023; Erdogan et al., 2025). Supervisor career support mentoring may facilitate employees’ resource-management process during career crafting and influence their assessment of their ability to cope with the environment. This, in turn, strengthens the impact of career crafting on their sense of control and subsequent career outcomes.

Specifically, supervisor career support mentoring provides employees with valuable learning opportunities and facilitates the development of professional skills (Gao et al., 2020; Zacher, 2016), giving them the resources needed to more effectively achieve their career goals. First, through career support mentoring, supervisors can reduce employees’ anxiety, foster positive expectations, and assist them in successfully navigating their careers, strengthening their self-assessment of their abilities and expectations for attainable career outcomes (Deng et al., 2023). This enhances the connection between career crafting, a sense of control, and subsequent career outcomes. Second, supervisor career support mentoring grants employees greater empowerment and autonomy (Erdogan et al., 2025). As employees enhance their sense of control through career crafting, they are more likely to receive understanding and support from their supervisors, which boosts the effectiveness of their career-crafting efforts. Additionally, such employees can better manage their emotions and psychological states, which helps enhance their career adaptability (P. C. Chang et al., 2023; Sunarjo et al., 2021). Career adaptability refers to an individual’s preparedness and resources for successfully coping with current and upcoming career-development tasks, career transitions, and personal setbacks (Federici et al., 2021; P. C. Lee et al., 2021; Tavitiyaman et al., 2025; T. Wang et al., 2024). Supervisor career support mentoring can improve employees’ career adaptability, helping them effectively manage career changes and challenges (P. C. Lee et al., 2021) and enhancing the effectiveness of their career crafting.

When employees perceive a higher level of supervisor career support mentoring, they typically gain more resources and freedom to engage in career crafting and are more confident that their actions will yield the desired results (Deng et al., 2023; Erdogan et al., 2025), which further increases their sense of control. Conversely, when employees work under lower levels of supervisor career support mentoring, their ability to craft their careers effectively is limited, potentially weakening the link between career crafting, their sense of control, and subsequent career outcomes. In summary, higher levels of supervisor career support mentoring strengthen the impact of career crafting on employees’ sense of control, enhancing their career engagement and reducing their career turnover intentions.

**Hypothesis 3.**  *Supervisor career support mentoring moderates the indirect effects of career crafting on career engagement (H3a) and career turnover intentions (H3b) through a sense of control, such that the indirect effects are stronger when supervisor career support mentoring is higher (vs. lower).*

## 3. Methods

### 3.1. Sample and Procedure

This study recruited 275 frontline employees working in various hotels across China through the crowdsourcing platform, Credamo. Employment instability and limited career development opportunities in the hospitality sector highlight the importance of employees’ career development (Liu-Lastres et al., 2023). Credamo was chosen for its diverse participant pool (W. Li et al., 2024b), which strengthened the external validity of the study’s findings. Participants were required to meet three criteria: (1) full-time employment; (2) at least one year of full-time work experience since employees who did not meet these criteria might lack sufficient career crafting experience (J. Y. Lee et al., 2021); and (3) regular or occasional interaction with their supervisors. Before data collection, we provided participants with a detailed explanation of the research objectives and procedures, emphasizing the importance of honest responses. To mitigate social desirability bias, we ensured complete anonymity and strict confidentiality, with all data used solely for academic purposes (Podsakoff et al., 2024). Prior to conducting the survey, we invited 5 professors and 10 graduate students in the Organizational Behavior and Human Resource Management field to review the survey items in depth and identify any ambiguous wording or phrases, ensuring that participants could easily understand the survey questions.

To alleviate concerns regarding CMV and reverse causality, this study adopted a three-wave data-collection approach (Podsakoff et al., 2024). Based on previous research on the consequences of job crafting (W. Li et al., 2024a; Tims et al., 2016), we set a one-week time interval between each wave of measurement. Specifically, at Time 1, participants reported their career crafting, supervisor career support mentoring, and demographic variables. At Time 2 (one week after Time 1), they assessed their sense of control. At Time 3 (one week after Time 2), they reported their career engagement and career turnover intentions. All surveys were administered on Fridays between 2:00 p.m. and 6:00 p.m., and participants could only proceed to the next wave if they completed the previous one. Data quality was ensured through two attention-check questions, and responses that failed these checks were excluded from the analysis. To improve participant engagement, we implemented two strategies. First, we sent reminders to participants who did not complete the survey within two hours. Second, participants received USD 1.5 for each completed survey, with an additional USD 1.5 bonus for those who completed all three waves. During data collection, 43 participants either withdrew from the study or failed the attention checks, which resulted in a final sample of 232 respondents. Among them, 150 were male, and 82 were female, with an average age of 31.71 years (*SD* = 6.97) and an average job tenure of 8.56 years (*SD* = 6.58). Participants’ educational backgrounds were as follows: high school or below (22.0%), three-year college (70.3%), and undergraduate degree (7.8%).

### 3.2. Measures

The key variables were measured using validated scales with established reliability. In line with Brislin’s (1980) back-translation procedure, the original English scales were translated into Chinese to ensure accuracy and conceptual equivalence. For each variable, we employed a 7-point Likert scale (1 = *strongly disagree* to 7 = *strongly agree*).

#### 3.2.1. Career Crafting (T1)

We used an eight-item scale developed by Tims and Akkermans (2020) to assess career crafting. Sample items included “I assess for myself what I really value in my career” and “I make sure that significant persons in my work are up to date about my performance and results”. Cronbach’s α for the scale was 0.82.

#### 3.2.2. Supervisor Career Support Mentoring (T1)

To measure employees’ perceptions of supervisor career support mentoring, we utilized a six-item scale from Deng et al. (2023). An example item was “My supervisor helped me coordinate my professional goals”. Cronbach’s α for the scale was 0.90.

#### 3.2.3. Sense of Control (T2)

We assessed a sense of control with an eight-item scale developed by Sirola (2024). An example item was “I can do just about anything that I really set my mind to”. Cronbach’s α for the scale was 0.83.

#### 3.2.4. Career Engagement (T3)

We utilized a nine-item scale created by Hirschi et al. (2014) to evaluate career engagement. An example item was “Undertook things to achieve your career goals”. Cronbach’s α for the scale was 0.81.

#### 3.2.5. Career Turnover Intentions (T3)

We utilized a three-item scale developed by Baillod and Semmer (1994) and adapted by Barthauer et al. (2020) to measure career turnover intentions. An example item was “I frequently think about abandoning my current career track”. Cronbach’s α for the scale was 0.83.

#### 3.2.6. Control Variables

To account for potential confounding effects, this study included controls for employees’ age, gender, education level, and job tenure for their established associations with career engagement and career turnover intentions (Hirschi et al., 2014; Luksyte & Carpini, 2024; Xu et al., 2024). Age and gender are linked to individuals’ career orientations and may shape the effectiveness of their career-crafting efforts (Janssen et al., 2021). Furthermore, employees with longer job tenure are less inclined to exit their careers given their substantial investments in time and resources toward professional growth (Luksyte & Carpini, 2024). To assess the robustness of our findings, we also conducted supplementary analyses without including these control variables, following the recommendation of Bernerth and Aguinis (2016). The results remained stable and consistent with our theoretical predictions, reinforcing the validity of our conclusions.

## 4. Results

### 4.1. Preliminary Analyses

As all primary variables in this study were measured using self-reported questionnaires, we took both procedural and statistical steps to mitigate concerns related to CMV. As a procedural remedy, we adopted a three-wave survey design, temporally separating the measurement of independent variables, mediators, and dependent variables. Second, Harman’s single-factor test indicated that the first factor accounted for only 27.34% of the total variance—well below the 50% threshold—which suggests that CMV was not a substantial concern (S. J. Chang et al., 2020). Furthermore, we assessed convergent validity using composite reliability (CR) and average variance extracted (AVE). The CR values for career crafting, supervisor career support mentoring, sense of control, career engagement, and career turnover intentions were 0.78, 0.93, 0.89, 0.83, and 0.83, respectively, all exceeding the 0.70 threshold (Hair et al., 1998). Similarly, the AVE values for these constructs were 0.54, 0.80, 0.62, 0.61, and 0.63, respectively, all above the 0.50 threshold. These findings reveal that the core constructs examined in this study demonstrate strong convergent validity. Additionally, this study performed a collinearity test using the variance inflation factor (VIF). The VIF values for age, gender, education level, job tenure, career crafting, and supervisor career support mentoring were 7.98, 1.01, 1.21, 8.33, 1.26, and 1.30, respectively. All VIF values were below 10, which indicates that multicollinearity among the variables was not a concern (O’brien, 1983; Shibiti, 2020). This study assessed the autocorrelation of residuals in the model using the Durbin–Watson (DW) test. The results show that the DW value was 1.97 for the regression model concerning career engagement and 1.91 for the regression model concerning career turnover intentions. According to the DW test standards (ranging from 0 to 4), both models had DW values close to 2, indicating no significant autocorrelation among the residuals. This suggested high reliability in the results of regression analysis (Durbin & Watson, 1950).

Before testing the hypotheses, we conducted a confirmatory factor analysis (CFA) to assess the distinctiveness of the main constructs. We chose this approach because the item-to-sample ratio (34:232) was lower than the recommended 1:10 threshold (Kunce et al., 1975). As CFA is particularly sensitive to sample size, a small sample may lead to factor solution instability (Little et al., 2002; Marsh & Hocevar, 1988), making item parceling the preferred method in this case. Career crafting comprises two key dimensions: proactive career reflection and proactive career construction. Following prior research (H. Li et al., 2024), we created two parcels by averaging the scores of each dimension to serve as indicators of career crafting. For other constructs containing more than three items (i.e., supervisor career support mentoring, a sense of control, and career engagement), we applied the item-to-construct balance approach (Little et al., 2002). Specifically, we created three parcels for supervisor career support mentoring, four for a sense of control, and three for career engagement by pairing the highest and lowest factor loading items as indicators of each latent variable. As illustrated in Table 1, the hypothesized five-factor model demonstrated a good fit with the data (*χ*^2^ = 124.89, *df* = 80, and *χ*^2^*/df* = 1.56; CFI = 0.97, TLI = 0.97, RMSEA = 0.05, and SRMR = 0.04) and significantly outperformed alternative models, supporting the discriminant validity of the constructs. Table 2 displays the descriptive statistics, correlations, and reliability of the key variables in this study.

### 4.2. Hypothesis Testing

To test Hypotheses 1 and 2, we utilized the SPSS 26.0 PROCESS macro developed by Hayes (2018). We examined mediation effects using a bootstrapping procedure with 5000 resamples and 95% confidence intervals (CIs)—an approach widely regarded for producing robust estimates in mediation, moderation, and moderated mediation analyses (Tang et al., 2020; Woehler et al., 2021). As shown in Table 3, career crafting was positively associated with a sense of control (*β* = 0.33, *p* < 0.001), supporting Hypothesis 1. A sense of control was positively linked to career engagement (*β* = 0.25, *p* < 0.001) and negatively predicted career turnover intentions (*β* = −0.52, *p* < 0.001). The indirect effect of career crafting on career engagement through a sense of control proved statistically significant (*estimate* = 0.08; 95% CI [0.045, 0.139]). Similarly, the indirect effect of career crafting on career turnover intentions via sense of control was significant (*estimate* = −0.17; 95% CI [−0.274, −0.094]). Therefore, Hypotheses 2a and 2b were supported.

**Figure 2:**
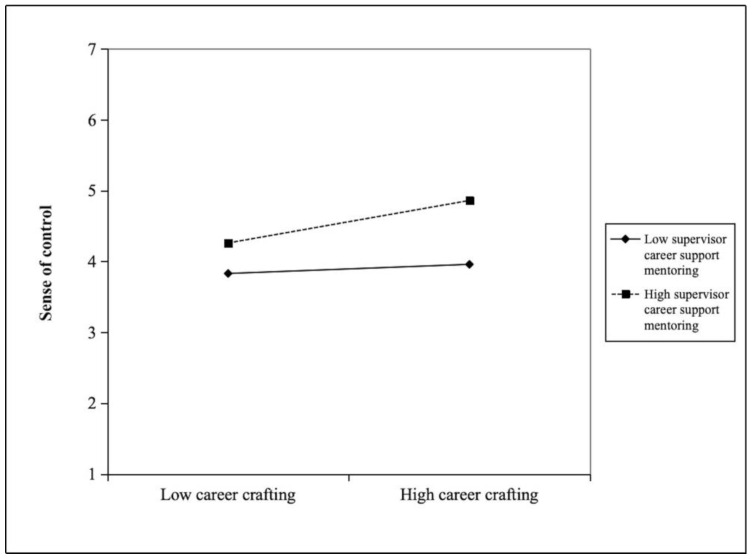
The moderating effect of supervisor career support mentoring on the relationship between career crafting and sense of control.

We conducted a hierarchical regression analysis to explore the moderating effect of supervisor career support mentoring on the relationship between career crafting and its outcomes. Prior to creating the interaction terms, we mean-centered both career crafting and supervisor career support mentoring to reduce the chance of multicollinearity. As displayed in Table 4, the interaction between career crafting and supervisor career support mentoring on a sense of control was significant and positive (*β* = 0.13, *p* < 0.01). A simple slope analysis further clarified the direction of this interaction. As illustrated in Figure 2, the relationship between career crafting and a sense of control was stronger when supervisor career support mentoring was higher (*B* = 0.37, *SE* = 0.08, *p* < 0.001) and weaker when supervisor career support mentoring was lower (*B* = 0.08, *SE* = 0.06, *p* = 0.20).

We then tested the moderated mediation effect of supervisor career support mentoring using the PROCESS macro (Hayes, 2018). As anticipated, the conditional indirect effect of career crafting on career engagement through a sense of control was significant when supervisor career support mentoring was higher (*estimate* = 0.06; 95% CI [0.021, 0.137]) but nonsignificant when supervisor career support mentoring was lower (*estimate* = 0.01; 95% CI [−0.008, 0.058]; see Table 5). The index of the moderated mediation effect did not contain zero (*estimate* = 0.02; 95% CI [0.004, 0.046]); thus, Hypothesis 3a was supported. Similarly, the conditional indirect effect of career crafting on career turnover intentions through a sense of control was stronger when supervisor career support mentoring was higher (*estimate* = −0.10; 95% CI [−0.226, −0.013]) but weaker when supervisor career support mentoring was lower (*estimate* = −0.02; 95% CI [−0.091, 0.018]). The index of the moderated mediation effect excluded zero (*estimate* = −0.04; 95% CI [−0.093, −0.001]); therefore, Hypothesis 3b was supported.

## 5. General Discussion

Building on COR theory and CCT, this study examined the underlying mechanisms and boundary conditions through which career crafting influences career engagement and career turnover intentions. Utilizing three-wave data collected from frontline employees in the Chinese hospitality industry, this study reveals that career crafting enhances employees’ sense of control, which, in turn, boosts their career engagement and lowers their career turnover intentions. Furthermore, it identifies supervisor career support mentoring as a critical contextual factor that moderates the relationship between career crafting and a sense of control. When employees perceive higher levels of supervisor career support mentoring, the positive impact of career crafting on their sense of control becomes more pronounced. This heightened sense of control further strengthens employees’ career engagement while reducing their career turnover intentions. In this section, we discuss the theoretical implications and practical contributions of this study.

### 5.1. Theoretical Implications

This study makes several important theoretical contributions. First, integrating COR theory and CCT (Hobfoll, 1989, 2001; Savickas, 2013) broadens our theoretical understanding of how career crafting affects career engagement and career turnover intentions, adding to the expanding body of research on career crafting. Previous research has focused on the impact of job crafting on career-related outcomes (Holman et al., 2024; S. Park & Park, 2023). However, while job crafting aims to improve the alignment between individuals and their current jobs (Harju et al., 2021; W. Li et al., 2024a), career crafting encompasses a broader perspective, involving proactive career planning not only within one’s current job but also across jobs, employers, occupations, and industries (J. Y. Lee et al., 2021; Leong et al., 2024; Tims & Akkermans, 2020). With the rapid advancement of AI and the deepening digital transformation, career paths are becoming increasingly fluid (Bankins et al., 2024), particularly in the hospitality industry (Leong et al., 2024). In response, scholars have called for empirical research on the impact of career crafting to improve the understanding of proactive career behaviors (J. Y. Lee et al., 2021; Tims & Akkermans, 2020). Addressing this call, this study supplements the limited research on career crafting by exploring its potential effects among frontline employees in the hospitality industry, filling gaps in our current understanding of career crafting and its implications.

Second, drawing on COR theory and CCT (Hobfoll, 1989, 2001; Savickas, 2013), we identify a sense of control as a key cognitive resource that mediates the relationship between career crafting and two critical outcomes: career engagement and career turnover intentions. Although previous studies have linked career crafting to various work-related and career outcomes (J. Y. Lee et al., 2021; Leong et al., 2024; Ni et al., 2024), the mechanisms by which it mitigates resource loss and promotes resource gain remain insufficiently explored. Specifically, employees who proactively engage in career crafting to obtain additional resources tend to experience a greater sense of control, which, in turn, enhances career engagement while lowering their career turnover intentions. Our findings integrate the literature on career crafting and sense of control within the COR and CCT frameworks, thereby broadening their applicability and providing new theoretical directions for future research. Furthermore, our findings offer compelling evidence that supervisor career support mentoring enhances the effectiveness of career crafting, clarifying the boundary conditions for its impact on a sense of control and subsequent career outcomes. According to COR theory, a supportive social environment helps employees acquire, protect, and sustain resources (Hobfoll et al., 2018). This study incorporated a key contextual factor to support this perspective while also addressing a previous call for research on the role of significant others—such as supervisors—in employees’ career-crafting processes (Tims & Akkermans, 2020). Supervisor career support mentoring encompasses skill development opportunities, professional network expansion, and access to challenging tasks (Deng et al., 2023). These forms of support strengthen the relationship between career crafting and a sense of control. In essence, high levels of supervisor career support mentoring amplify the positive effect of career crafting on a sense of control, increasing employees’ career engagement and reducing their career turnover intentions. By incorporating supervisor career support mentoring as a moderating variable, this study deepens our understanding of the conditions under which employees’ career crafting is more effective. This study draws on COR theory to explain proactive career behaviors among frontline employees in the high-turnover hospitality industry. By linking career crafting to resource acquisition and preservation and integrating it with organizational support systems—specifically, supervisor career support mentoring—we offer a structurally distinctive and practically relevant model for sustainable career development.

Finally, this study expands the conceptual framework of a sense of control by linking it to career crafting as a key antecedent. Thus far, empirical research has primarily examined the causes of employees’ sense of control or loss of control from the perspectives of workplace contextual factors and individual characteristics (A. Li et al., 2021; Mell et al., 2022). This study makes two substantial contributions to the literature. First, it demonstrates that career crafting has a strong positive impact on a sense of control. As a proactive, bottom-up career behavior, career crafting broadens the antecedents of a sense of control by incorporating proactive workplace behaviors into the discussion. Second, unlike previous studies that have focused on either contextual or individual factors in isolation, this study introduces supervisor career support mentoring as a moderating variable, examining the interaction between supervisors and employees in shaping employees’ sense of control through career crafting. By doing so, it enhances the understanding of the key factors influencing employees’ sense of control in the workplace. Additionally, this study employs a multi-wave data-collection approach to investigate the effects of career crafting among frontline employees in the hospitality industry. This research design effectively minimizes the risks of common method bias and reverse causality (Podsakoff et al., 2024). By utilizing a three-wave survey, the study offers a more comprehensive and precise understanding of the mechanisms underlying career crafting among frontline employees.

### 5.2. Practical Implications

This study offers valuable insights into organizational management strategies in the hospitality sector. First, our findings demonstrate that career crafting enhances employees’ career engagement while reducing their career turnover intentions, offering organizations and managers a deeper understanding of how to effectively leverage career crafting. Organizations may be concerned that encouraging employees’ career development could be counterproductive, potentially increasing employee turnover. However, our results indicate that investing in employees’ career development benefits organizations by strengthening career engagement and lowering career turnover intentions. Moreover, employees increasingly expect organizations to offer career development opportunities that support their professional growth (J. Y. Lee et al., 2021; Tims & Akkermans, 2020). Therefore, organizations should actively facilitate and support employees’ career-crafting efforts. Although career crafting is inherently a self-initiated behavior, employees may not always be fully aware of its benefits (Janssen et al., 2021; Leong et al., 2024). Organizations can foster and promote career crafting through targeted interventions, such as training programs or challenging job assignments (Janssen et al., 2021). For instance, organizations can conduct regular career-crafting workshops to help employees identify their strengths, interests, and career aspirations, guiding them toward practical strategies to align their career trajectories with their personal interests and competencies. Additionally, employees should take an active role in shaping their careers. Those experiencing uncertainty about their career path can obtain support through career counseling. Career counselors can assist employees in evaluating the feasibility of different career options and analyzing the costs and benefits of achieving career goals. They can also provide actionable recommendations to enhance the effectiveness of career crafting.

Second, this study identified a sense of control as a critical mediator in the relationships between career crafting and career engagement, as well as career crafting and career turnover intentions, highlighting it as a key area for organizational intervention. Organizations and managers should prioritize strategies that enhance employees’ sense of control. For example, organizations can offer personalized skill development programs, such as communication and job-specific training, to help employees build confidence in their abilities and strengthen their sense of control (A. Li et al., 2021). Furthermore, our findings indicate that supervisor career support mentoring amplifies the impact of career crafting on a sense of control and its subsequent effects on career engagement and career turnover intentions. Therefore, organizations should systematically establish and strengthen career support mechanisms to ensure that supervisors provide effective career guidance and support. Specifically, organizations can institutionalize career mentoring by integrating career support into supervisors’ core management responsibilities. For instance, they can implement regular one-on-one career development meetings (weekly or monthly) to help supervisors gain deeper insights into employees’ career challenges and psychological concerns while providing personalized career advice and resources (Erdogan et al., 2025). Additionally, supervisors should act as career enablers, helping employees navigate an increasingly dynamic work environment. This includes fostering open discussions about industry trends and organizational changes, encouraging continuous skill development, and aligning employees’ career trajectories with organizational needs. These initiatives would not only enhance the positive impact of career crafting but also contribute to the sustainable development of both employees and organizations.

### 5.3. Limitations and Future Directions

This study has several limitations that should be addressed in future research. First, while the three-wave data collection method strengthened the robustness of the findings (Podsakoff et al., 2024), all key variables—career crafting, supervisor career support mentoring, a sense of control, career engagement, and career turnover intentions—were measured through employee self-reports. While self-reports effectively capture participants’ subjective perceptions and psychological experiences, they make establishing causal relationships difficult. To address concerns about reverse causality, we calculated Akaike’s Information Criterion (AIC) and Bayesian Information Criterion (BIC) for both the proposed model and alternative models, following previous research (Yam et al., 2018). The model with the lowest AIC and BIC values is considered to fit the data best (Kline, 2023). The results show that the model for Hypothesis 1 (career crafting → sense of control) has lower AIC and BIC values (AIC = 498.83, BIC = 522.96) compared to the reverse causality model (sense of control → career crafting: AIC = 1078.48, BIC = 1123.29). Additionally, the model for Hypothesis 2a (career crafting → sense of control → career engagement) has lower AIC and BIC values (AIC = 840.50, BIC = 592.20) compared to the reverse causality model (career engagement → sense of control → career crafting: AIC = 1788.06, BIC = 1846.65). Similarly, the model for Hypothesis 2b (career crafting → sense of control → career turnover intentions) shows lower AIC and BIC values (AIC = 1229.19, BIC = 1280.89) compared to the reverse causality model (career turnover intentions → sense of control → career crafting: AIC = 1439.02, BIC = 1497.62). While these findings strengthen the validity of the directional effects, we recommend that future research incorporate objective (e.g., turnover rates) and multi-source (e.g., supervisor ratings of employee career engagement) data and employ experimental or quasi-experimental designs to enhance the rigor of causal inferences and improve the findings’ external validity.

Second, drawing on COR theory and CCT, this study investigated how career crafting influences employees’ career engagement and career turnover intentions through their sense of control. Given the current lack of research on the mediating mechanisms through which career crafting operates, future studies could explore other mediators to offer a more comprehensive explanation of its effects. For example, from a self-determination theory perspective (Deci et al., 2017), career crafting may fulfill employees’ basic psychological needs, fostering harmonious passion, which may, in turn, boost career engagement and reduce career turnover intentions. Future research could integrate multiple theoretical perspectives to investigate the underlying mechanisms through which career crafting affects career outcomes, expanding our understanding of its broader impact. Additionally, this study primarily focused on the moderating role of supervisor career support mentoring in the link between career crafting and a sense of control. Future research could further explore other contextual or individual factors that may support or hinder employees’ engagement in career crafting. For instance, spousal support may provide employees with resources to better manage their careers (Ocampo et al., 2018), strengthening the connection between career crafting and a sense of control. Therefore, future research should aim to identify the contextual and individual boundary conditions of career crafting to provide a more precise theoretical foundation for career-management strategies. Finally, this study’s sample included frontline employees from multiple hotels in China, providing some degree of representativeness for the Chinese hospitality industry. However, the applicability of these findings to the hospitality industry in other countries requires further investigation. We look forward to future research that gathers samples from diverse cultural contexts to strengthen the generalizability of our conclusions.

## Figures and Tables

**Table 1:** Results of confirmatory factor analyses.

Model	*χ* ^2^	*df*	*χ*^2^/*df*	CFI	TLI	RMSEA	SRMR
1. Five-factor model	124.89	80	1.56	0.97	0.97	0.05	0.04
2. Four-factor model:	201.37	84	2.40	0.93	0.92	0.08	0.06
CC + SOC							
3. Three-factor model:	401.94	87	4.62	0.82	0.78	0.13	0.09
CC + SOC + SCSM							
4. Two-factor model:	618.19	89	6.95	0.70	0.65	0.16	0.11
CC + SOC + SCSM + CTI							
5. One-factor model:	761.97	90	8.47	0.62	0.56	0.18	0.11

Note: *N* = 232. CC = career crafting; SOC = sense of control; SCSM = supervisor career support mentoring; CE = career engagement; CTI = career turnover intentions.

**Table 2:** Descriptive statistics, correlations, and reliability.

Variable	*Mean*	*SD*	1	2	3	4	5
1. Career crafting (T1)	4.95	0.83	0.82				
2. Supervisor career support mentoring (T1)	5.08	1.09	0.44 **	0.90			
3. Sense of control (T2)	5.15	0.75	0.35 **	0.46 **	0.83		
4. Career engagement (T3)	5.78	0.56	0.36 **	0.45 **	0.41 **	0.81	
5. Career turnover intentions (T3)	2.93	1.25	−0.23 **	−0.46 **	−0.36 **	−0.49 **	0.83

Note: *N* = 232. ** *p* < 0.01. The reliability of the scales is noted in the diagonals.

**Table 3:** Indirect effects of a sense of control.

**Direct Effect**	***B***	***SE***
Career crafting → Sense of control	0.33 ***	0.05
Sense of control → Career engagement	0.25 ***	0.05
Sense of control → Career turnover intentions	−0.52 ***	0.11
Career crafting → Career engagement	0.16 ***	0.04
Career crafting → Career turnover intentions	−0.18	0.10
**Indirect Effect**	***Estimate***	**95% CI**
Career crafting → Sense of control → Career engagement	0.08	[0.045, 0.139]
Career crafting → Sense of control → Career turnover intentions	−0.17	[−0.274, −0.094]

Note: *** *p* < 0.001. CI = confidence interval. Bootstrap sample size = 5000.

**Table 4:** Unstandardized coefficients of the moderation effect.

Variables	Sense of Control		
	Model 1	Model 2	Model 3
Constant	4.11 *** (0.49)	4.27 *** (0.42)	4.23 *** (0.41)
*Controls*			
Age	−0.24 * (0.10)	−0.32 *** (0.09)	−0.33 *** (0.09)
Gender	0.04 * (0.02)	0.03 * (0.02)	0.03 * (0.02)
Education	0.03 (0.10)	0.10 (0.09)	0.10 (0.08)
Job tenure	−0.04 (0.02)	−0.02 (0.02)	−0.03 (0.02)
*Predictor*			
Career crafting (A)		0.17 ** (0.06)	0.22 *** (0.06)
Supervisor career support mentoring (B)		0.27 *** (0.04)	0.31 *** (0.04)
A × B			0.13 ** (0.04)
*R* ^2^	0.05	0.30 ***	0.34 **
Δ*R*^2^		0.25	0.04
*F value*	2.84	16.15 ***	16.26 ***

Note: * *p* < 0.05; ** *p* < 0.01; *** *p* < 0.001.

**Table 5:** Conditional indirect effect of career crafting on career engagement and career turnover intentions via sense of control.

Moderator	Level	Outcome Variables	Indirect Effect	Boot SE	95% CI
Supervisor career support mentoring	Low	Career engagement	0.01	0.02	[−0.008, 0.058]
	Medium		0.04	0.02	[0.011, 0.089]
	High		0.06	0.03	[0.021, 0.137]
	Dif		0.05	0.03	[0.008, 0.110]
	Low	Career turnover intentions	−0.02	0.03	[−0.091, 0.018]
	Medium		−0.06	0.03	[−0.143, −0.009]
	High		−0.10	0.05	[−0.226, −0.013]
	Dif		−0.08	0.05	[−0.207, −0.002]

Note: Bootstrap sample size = 5000.

## Data Availability

The data presented in this study are available upon request from the corresponding author.
